# A second‐generation CD38‐CAR‐T cell for the treatment of multiple myeloma

**DOI:** 10.1002/cam4.5818

**Published:** 2023-04-11

**Authors:** Hongwen Li, Jing Li, Jiazhuo Wu, Zhuangzhuang Shi, Yuyang Gao, Wenting Song, Jiwei Li, Zhaoming Li, Mingzhi Zhang

**Affiliations:** ^1^ Department of Oncology The First Affiliated Hospital of Zhengzhou University Zhengzhou China; ^2^ State Key Laboratory of Esophageal Cancer Prevention and Treatment and Henan Key Laboratory for Esophageal Cancer Research The First Affiliated Hospital of Zhengzhou University Zhengzhou China; ^3^ Department of Medical Oncology, State Key Laboratory of Oncology in South China, Collaborative Innovation Center for Cancer Medicine Sun Yat‐sen University Cancer Center Guangzhou China; ^4^ Academy of Medical Sciences of Zhengzhou University Zhengzhou China

**Keywords:** CD38, chimeric antigen receptor, immunotherapy, multiple myeloma

## Abstract

**Background:**

Multiple myeloma (MM) is an aggressive plasma cell malignancy, causing a number of deaths worldwide every year. Chimeric antigen receptor (CAR) transduced T‐cell therapy has been a promising immunotherapy against hematological malignancies.

**Methods:**

In this study, we developed a second‐generation CAR construct and generated CAR‐T cells targeting CD38 molecule. Then effects of CAR‐T cells against MM cell lines were evaluated.

**Results:**

CD38‐CAR‐T cells showed higher cytotoxicity to MM cell lines and primary MM cells than that of control T cells in vitro. Over 50% MM1.s and RPMI8226 cells were killed by CAR‐T cells even at effector to target ratio of 1:100. CAR‐T cells also showed an enhanced cytotoxicity against primary MM cells. CAR‐T cells could be activated and produced a variety of cytokines in a target‐dependent manner. In vivo test indicated that CAR‐T cells also showed significant antitumor effect on xenograft mice models.

**Conclusion:**

These results indicated a promising therapeutic strategy of CD38‐CAR‐T cells against MM.

## Graphical abstract

A second generation CD38‐CAR‐T cell was generated. CD38‐CAR‐T cells showed significant high cytotoxicity to MM cells both in vitro and in vivo. CD38‐CAR‐T cells can be activated by MM cells and produce inflammatory cytokines in a target‐dependent manner. In vivo test indicated that CAR‐T cells also showed significant anti‐tumor effect on xenograft mouse models.

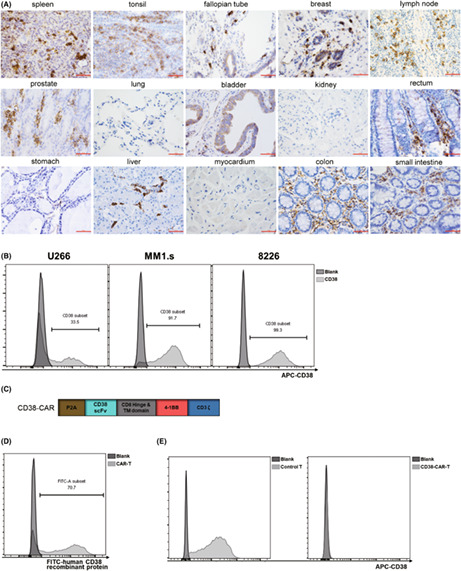

## 1 INTRODUCTION

Multiple myeloma (MM), an aggressive malignancy derived from plasma cells, remains the second most common hematological tumor in the world. Melphalan and prednisone used to be the first‐line therapy for MM. However, the 5‐year survival rate of MM patients was only 34.5%–49.6%.^1^ In recent years, novel therapeutic methods, including proteasome inhibitors, immunomodulatory drugs, and targeted immunotherapy agents, have contributed to improved progression‐free survival and median overall survival.^2^ Nonetheless, drug resistance and poor prognosis of patients with relapse or refractory MM (RRMM) remain great challenges for hematologists and oncologists.^3^ Thus, it is necessary to develop novel therapies to reduce the risk of recurrence and improve patient survival.

A new strategy, CAR‐T‐cell immunotherapy has shown tremendous potential in the treatment of hematological malignancies over the past decade.^1^, ^2^ CAR‐T cells targeting different molecules such as CD19, CD20, CD22, and BCMA have been developed and investigated for the administration of leukemia, lymphoma, and MM.^3^, ^4^, ^5^, ^6^, ^7^

CD38 plays an important role in pathogenesis and prognosis of human immunodeficiency virus infection and chronic lymphocytic leukemia.^8^ Due to its high expression on the surface of MM cells, CD38 has been considered as a promising immunotherapy target for MM.^8^, ^9^, ^10^, ^11^, ^12^, ^13^ Anti‐CD38 monoclonal antibodies such as daratumumab and isatuximab have been evaluated in the treatment of MM. However, the overall response rate (ORR) was only 36% in MM patients treated with daratumumab as a single agent.^12^ While the ORR is 50%–60% in relapsed MM patients treated with isatuximab, lenalidomide, and dexamethasone.^17^ There have been studies of CAR‐T cells targeting CD38 molecule alone or in combination with B‐cell maturation antigen (BCMA).^18^, ^19^, ^20^, ^21^ Clinical trials (NCT03464916 and NCT03767751) are being conducted to evaluate the efficacy of these CAR‐T cells in MM patients.

In this study, we generated a second‐generation CD38‐CAR‐T cells and assessed their antitumor effect in vitro and in vivo. We aimed to provide an immunotherapy for clinical practice and improve the treatment efficiency of myeloma.

## 2 MATERIALS AND METHODS

### 2.1 Cell lines

MM cell lines, U266, MM1.s, and RPMI8226 were purchased from iCell Bioscience Inc. (Shanghai). U266 and MM1.s cells were cultured in RPMI‐1640 medium supplemented with 15% FBS (Gibco) and antibiotics (100 U/mL penicillin and 100 μg/mL streptomycin). RPMI8226 cells were cultured in IMDM medium supplemented with 20% FBS and antibiotics. Cells were cultured in the incubator (Thermo Fisher) at 37°C and 5% CO_2_.

### 2.2 Immunohistochemistry (IHC) staining

Tissues were fixed in formalin, decalcified, and paraffin‐embedded. Following antigen retrieval, sections were blocked with 0.3% H_2_O_2_ in methanol. Then they were boiled in citrate buffer and blocked with serum‐free protein blocking solution. The sections were incubated with anti‐CD38 (Servicebio, GB13014) antibody overnight at 4°C. Then they were counterstained with hematoxylin, dehydrated through a graded alcohol series, cleared in xylene, and covered with coverslips. The percentage of CD38‐positive cells was scored as follows: 0 point represents negative expression (positive cells <5%), 1 point represents weakly positive expression (positive cells ranged from 5% to <25%), 2 points represent positive expression (positive cells ranged from 25% to <50%), 3 points represent strongly positive expression (positive cells ≥50%).

### 2.3 CAR construct and generation of lentiviral particles

CAR sequence included a single chain variable fragment (scFv) format and a signaling sequence. The sequences of scFv domain of CD38 antibodies were synthetically produced by GENEWIZ (Suzhou). The scFv sequence was cloned into pEF‐MCS‐P2A‐EGFP vector with a CD8a transmembrane domain, a 4‐1BB costimulatory domain, and a CD3ζ sequence.

The pRSV‐Rev, pLP‐VSVG, pCMV‐Gag‐Pol vectors, and CAR construct vectors were transfected using PEI transfection reagent (Sigma) into 293 T cells, cultured in DMEM medium supplemented with 10% FBS and antibiotics. Seventy‐two hours after transfection, the supernatants containing lentiviral particles were collected and concentrated using an ultrafiltration device (Merck Millipore).

### 2.4 Generation of CAR‐T cells

Informed consent was obtained before the study started, and the study was approved by the Institutional Research Ethics Committee of the First Affiliated Hospital of Zhengzhou University (Lot No. 2022‐KY‐1062). T cells were isolated from peripheral blood mononuclear cells (PBMCs) of healthy donors using magnetic activated cell sorting (MACS) method with CD3 Dynabeads (Miltenyi Biotec). Then they were cultured and expanded in X VIVO‐15 medium (LONZA) supplemented with 200 IU/mL IL‐2 for 3–5 days. T cells were transduced by incubation of CAR lentivirus (CD38‐CAR‐T) or vector lentivirus (Control T). Forty‐eight hours later, transfection rate of T cells was detected by FACS with recombinant human CD38 protein (FITC‐labeled, ACROBiosystems).

### 2.5 Cell‐mediated cytotoxicity assay

CD38‐CAR‐T or control T cells (effector) were cocultured with MM cell lines (target cells) at E:T (effector to target) ratio of 2:1, 1:1, 1:2, and 1:4 for 6 h. Then the cytotoxicity of CAR‐T cells was determined by measuring lactate dehydrogenase (LDH) in the supernatant. LDH was detected by CytoTox 96® Non‐Radioactive Cytotoxicity Assay kit (Promega).

To evaluate the cytotoxicity of CAR‐T cells at lower E:T ratios, T cells were activated by CD3/28 dynabeads (Gibco) for 3 days and then transduced with CD38‐CAR lentivirus. MM cell lines were labeled by CFSE and then cocultured with CD38‐CAR‐T or control T cells at a serial E:T ratios of 1:10, 1:20, 1:50, and 1:100 for 24 h. Then cells were stained with Annexin V and PI (Keygen Biotech) and analyzed by a FACS Calibur flow cytometer. Cytotoxicity of CD38‐CAR‐T cells against autologous PBMCs and primary tumor cells isolated from bone marrow in MM patients was also evaluated. PBMCs or primary tumor cells were co‐incubated with CAR‐T and control T cells at a E:T ratio of 1:1 or 1:10 for 24 h and analyzed by a FACS Calibur flow cytometer.

### 2.6 Detection of T‐cell activation and proliferation

To detect T‐cell activation biomarkers, CD38‐CAR‐T cells or control T cells were incubated with MM cell lines (treatment group) at a ratio of 1:10 or medium (blank group) for 24 h. Then biomarkers in different subsets of T cells were detected by a FACS Calibur flow cytometer with anti‐CD4, CD8, CD25, CD69, and HLA‐DR fluorescein‐conjugated antibodies (BD Biosciences).

To evaluate the proliferation of T cells, CD38‐CAR‐T and control T cells were labeled by CFSE and then incubated with MM cell lines (treatment group) at a ratio of 1:20 or medium (blank group) for 72 h. Then the FITC fluorescence intensity of T cells was detected and analyzed by a FACS Calibur flow cytometer (BD Biosciences).

### 2.7 Cytokine measurements

Cytokine bead array (CBA) was used to detect the cytokines produced by CAR‐T or control T cells. 2 × 10^4^ CAR‐T or control T cells were cocultured with 2 × 10^5^ target MM cells (treatment group) or medium (blank group) in 200 μL volume for 24 h. Then 50 μL cell supernatant was incubated with specific capture beads (IL‐2, IL‐3, IL‐8, IL‐9, GM‐CSF, granzyme B, TNF‐α, and IFN‐γ) and PE‐labeled detection antibodies for 2 h. After washing, beads were analyzed by a standardized flow cytometry assay (BD Biosciences).

### 2.8 In vivo experiment

NSG mice (3–4 weeks old, female, 15–20 g) used in this study were obtained from Shanghai Model Organisms Center. A total of 5 × 10^6^ RPMI8226‐Luciferase cells in 200 μL medium were mixed with isopycnic matrigel (Corning Incorporated) and implanted subcutaneously into the abdominal wall of mice. Ten days after tumor implantation, treatment groups of mice (*n* = 6 per group) received tail intravenous injection of CAR‐T or control T cells at Days 12 and 17 with a total of 5 × 10^6^ T cells per mice (about 2 × 10^6^ transduced cells). Mice in blank group were untreated. Animal experiments were conducted in accordance with the Declaration of Helsinki and approved by the Institutional Research Ethics Committee of the First Affiliated Hospital of Zhengzhou University (Lot No. 2022‐KY‐1062). Mice were given D‐Luciferin (150 mg/kg, i.p.) and anesthetized with isoflurane. After 5 min, luminescence was detected using the in vivo imaging system (IVIS) and the intensity was quantitated and normalized by the Living Image software (PerkinElmer).

### 2.9 Statistical analysis

Statistical analysis was performed using GraphPad Prism version 5.0 (GraphPad Software, Inc.). Results were reported as the mean ± standard deviation or median and interquartile range for repeated measurements. The *t*‐test and Mann–Whitney test were applied to assess differences between normally distributed samples and non‐normally distributed samples, respectively. *p* < 0.05 was considered statistically significant.

## 3 RESULTS

### 3.1 Expression of CD38 in different tissues and MM cell lines

**FIGURE 1:**
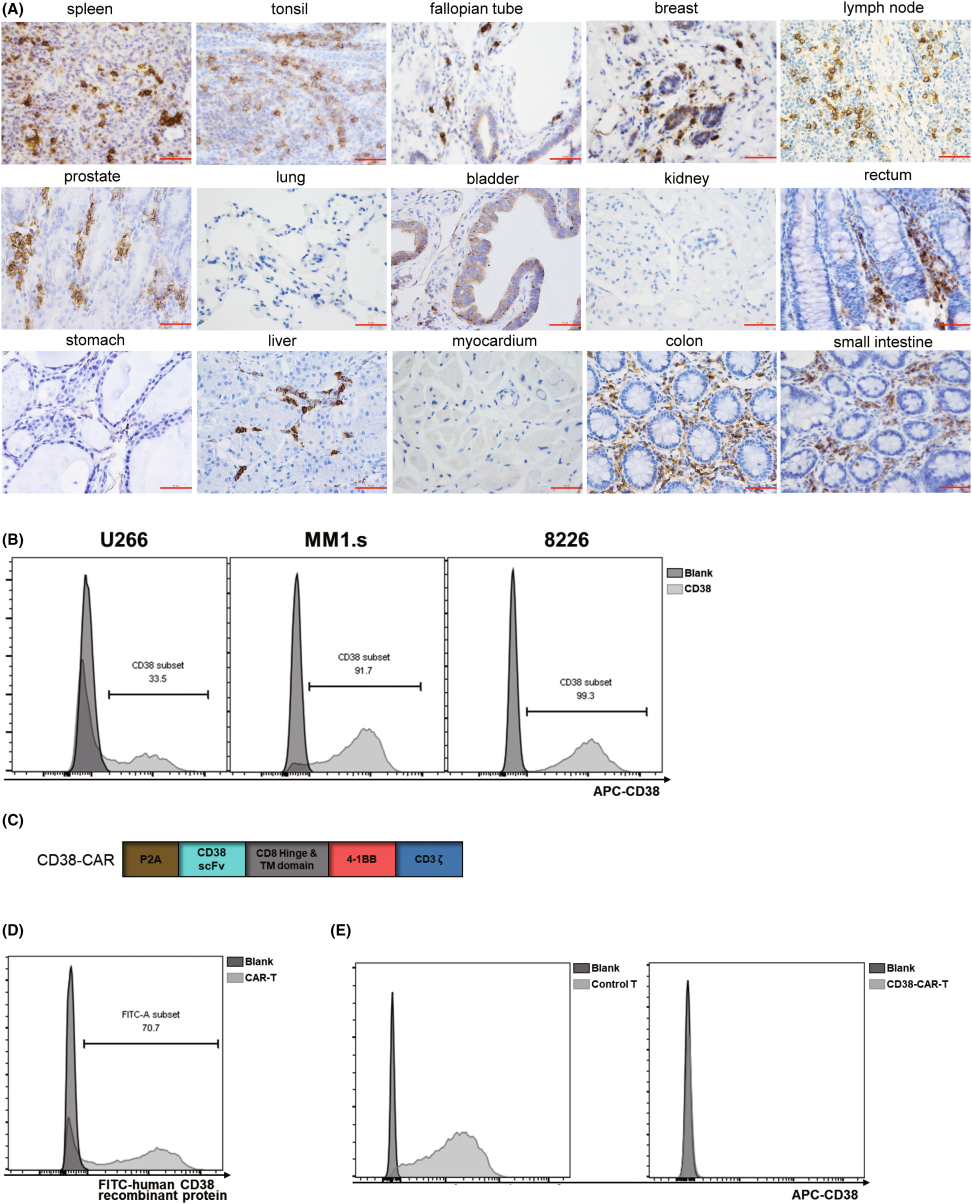
Expression of CD38 in human tissues and MM cells. (A) Expression of CD38 in a variety of tissues. (B) Expression of CD38 in U266, MM1.s, and RPMI8226 cells. (C) Construct of CD38‐CAR. (D) CAR expression was detected with FITC‐labeled human CD38 protein. (E) Expression of CD38 on control T cells and CD38‐CAR‐T cells.

The expression of CD38 was detected in 178 tissues. Spleen, tonsil, fallopian tube, and breast tissue showed strongly positive expression of CD38. Prostate, lung, bladder, kidney, and rectum tissues showed varying degrees of CD38 expression (Table 1, Figure 1A).

**TABLE 1:** CD38 expression in different tissues.

Tissue	Number of cases	CD38 positivity score			
		3	2	1	0
Spleen	3	3			
Tonsil	10	3	7		
Oviduct	14	5	5	2	2
Mammary gland	10	4	2	1	3
Lymph node hyperplasia	10		5	1	4
Prostate	2	1			1
Lung	8	2	3		3
Bladder	4	2			2
Kidney	10		5	3	2
Rectum	6	2			4
Gallbladder	11		3	4	4
Uterus	15		4	2	9
Ovary	6		1	2	3
Thyroid	10		1	2	7
Esophagus	6		1		5
Appendix	7		1		6
Stomach	14			1	13
Liver	8				8
Myocardium	2				2
Adrenal gland	1				1
Duodenum	4				4
Colon	7				7
Pancreas	5				5
Small intestine	5				5

MM cell lines, U266, MM1.s, and RPMI8226, were used as target cells in this study. FACS detection showed heterogeneous expression of CD38 on the three MM cell lines (Figure 1B). The percentage of CD38^+^ subgroup was lowest in U266 cells (33.6%), while in MM1.s and 8226 cells it was over 90% (91.2% and 99.3%, respectively).

### 3.2 Generation of CD38‐CAR‐T cells

The anti‐CD38 scFv sequence is based on a CD38 antibody with binding affinity comparable to daratumumab.^14^ It was cloned into pEF‐MCS‐P2A‐EGFP vector (Figure 1C). T cells were isolated and transduced with CD38‐CAR lentivirus. The transduction efficiency was detected using FITC‐labeled human CD38 protein (Figure 1D).

CD38 is a T‐cell activation marker and involved in chemotaxis, development of T cells, and humoral immune responses.^15^, ^16^ However, transduced CAR‐T cells lost the expression of CD38 molecule (Figure 1E). Therefore, we performed a variety of experiments to assess the function of CD38‐CAR‐T cells.

### 3.3 Target‐dependent activation and proliferation of CAR‐T cells

**FIGURE 2:**
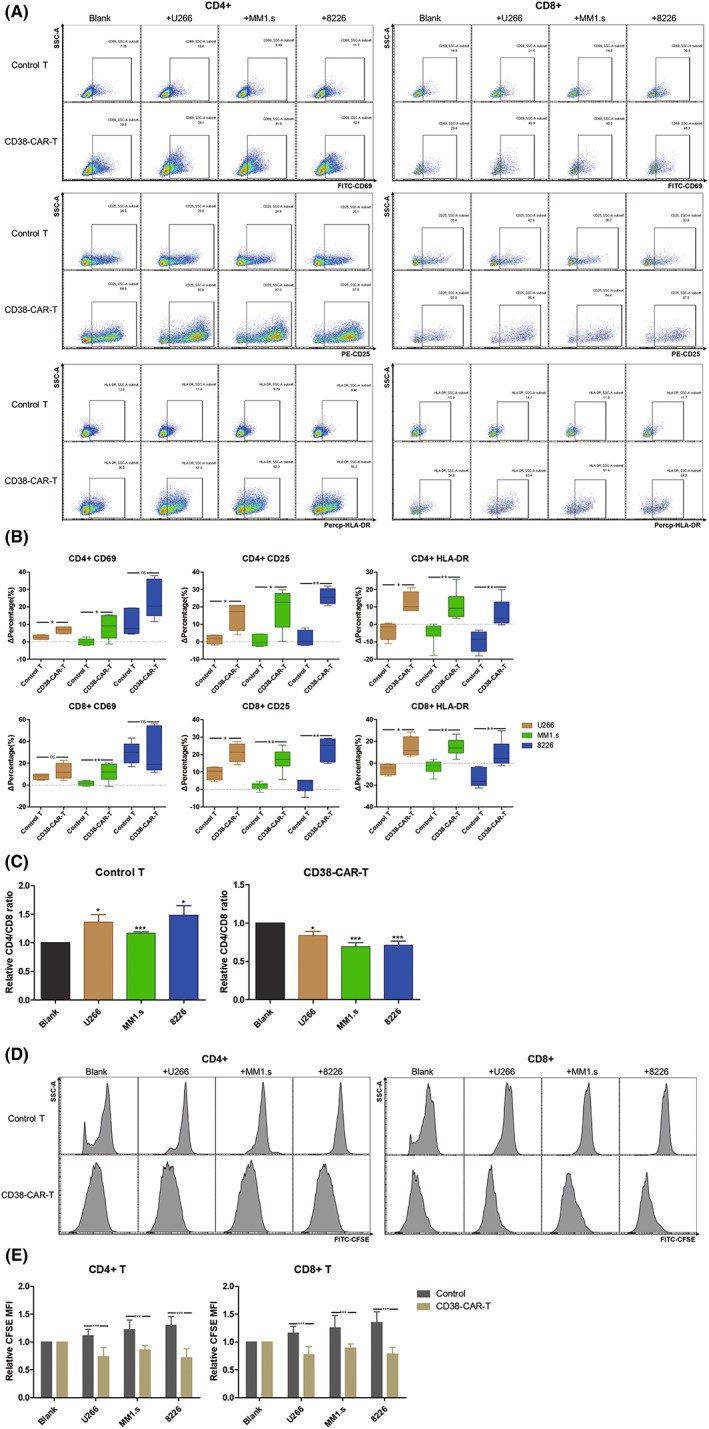
Activation and proliferation of CAR‐T cells. (A) Activation marker (CD69, CD25, and HLA‐DR) expression on CD4^+^ and CD8^+^ subgroups of T cells were detected by FACS. (B) Statistical results of activation markers expression on T cells. ΔPercentage is equal to the difference between the proportion of positive subpopulation of T cells in the treatment group and blank group. (C) Difference in the CD4/CD8 ratio of CD38^−^ CAR‐T cells and T cells stimulated by MM cells. Relative CD4/CD8 ratio = (CD4/CD8 ratio of treatment group)/(CD4/CD8 ratio of blank group). (D) Proliferation of CD38‐CAR‐T cells and control T cells stimulated by MM cells was detected by CFSE fluorescence intensity. (E) Statistical results of mean fluorescence intensity (MFI) of CD4^+^ and CD8^+^ subgroups of T cells. Lower MFI represent more rapid cell proliferation. Relative MFI = MFI of treatment group/MFI of blank group. *p* < 0.05 was considered statistically significant. ns, not significant; *p < 0.05; **p < 0.01; ***p < 0.001.

Stimulation of T cells may lead to the alteration of cellular activation surface markers, including early (CD69) and late (CD25 and HLA‐DR) markers.^17^ To investigate the target‐dependent activation of T cells, we assessed the expression of CD69, CD25, and HLA‐DR on both CD4^+^ and CD8^+^ subsets of CD38‐CAR‐T and control T cells. Data showed that except CD69 expression in CD8^+^ subgroup of CAR‐T and control T cells cocultured with U266 or 8266, expression of CD69, CD25, and HLA‐DR in both CD4^+^ and CD8^+^ subgroups of CAR‐T cells increased significantly compared with these markers in control T cells (Figure 2A,B).

There are differences in the CD4/CD8 ratio of CAR‐T cells and control T cells stimulated by MM cells. After 24 h cocultivation with MM cell lines, CD4/CD8 ratio decreased in CAR‐T cells and increased in control T cells (Figure 2C).

To evaluate T‐cell proliferation stimulated by MM cells, CFSE‐labeled CAR‐T and control T cells were cocultured with MM cell lines for 72 h. Then the CFSE fluorescence intensity of T cells was detected by FACS. T‐cell proliferation was determined by the decreasing CFSE fluorescence intensity. Results indicated that both CD4^+^ and CD8^+^ subgroups of CAR‐T cells proliferated significantly more than control T cells in response to MM cells (Figure 2D,E).

### 3.4 Cytotoxicity of CAR‐T cells against MM cells

**FIGURE 3:**
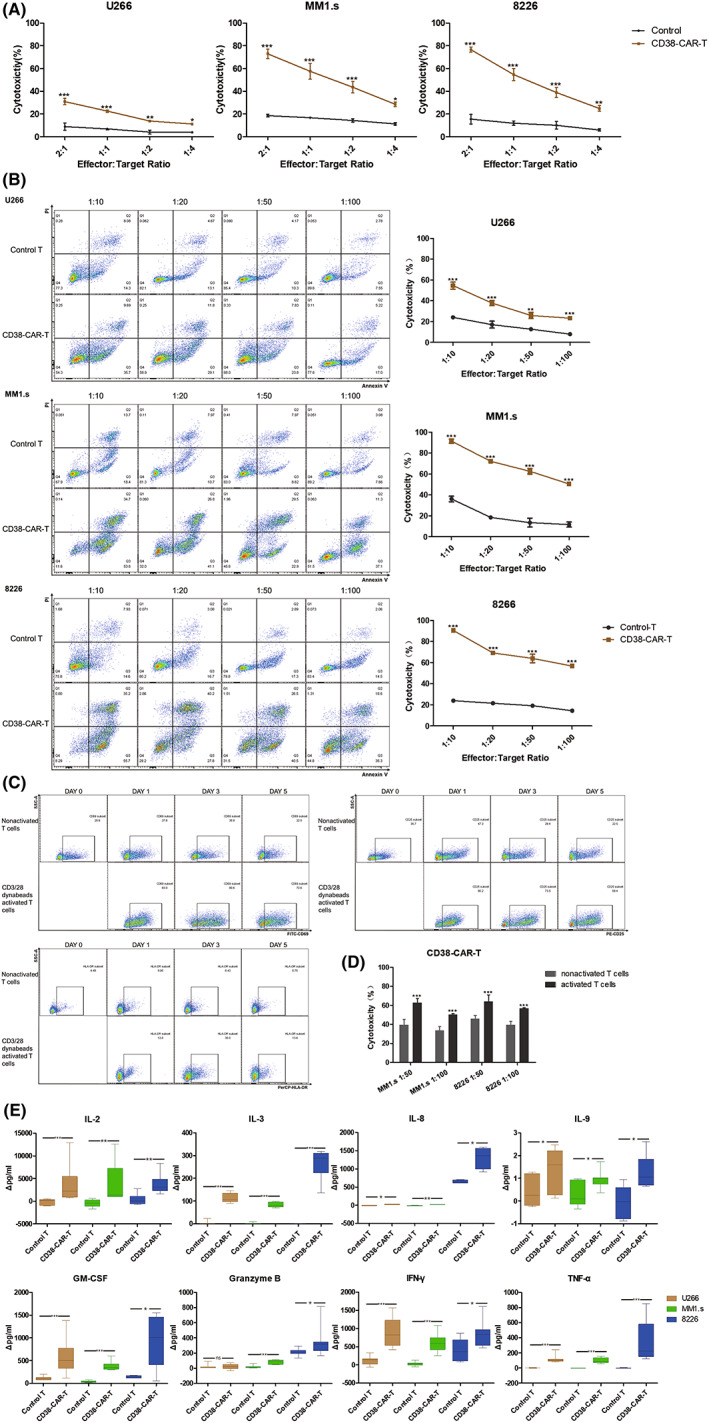
Cytotoxicity and cytokine‐releasing CAR‐T cells. (A) CD38‐CAR‐T or control T cells were coincubated with MM cell lines for 6 h. The cytotoxic effect of CD38‐CAR‐T and control T cells was evaluated by detecting LDH released by tumor cells. (B) CD38‐CAR‐T or control T cells were co‐incubated with CFSE‐labeled MM cell lines for 24 h. Cells were stained with Annexin V and PI to evaluate the cytotoxicity of T cells. (C) Expression of CD69, CD25, and HLA‐DR in nonactivated T cells and CD3/28 dynabeads activated T cells. (D) Differences between the cytotoxicity of CD38‐CAR‐T cells generated from nonactivated T cells and CD3/28 dynabeads activated T cells. (E) Cytokines released by CD38‐CAR‐T cells and control T cells were detected using the CBA kit. Δpg/mL refers to the difference between cytokine concentration in the treatment group and the blank group. *p* < 0.05 was considered statistically significant. ns, not significant; *p < 0.05; **p < 0.01; ***p < 0.001.

LDH released by lysed tumor cells was detected to evaluate the short‐time cytotoxicity of CAR‐T cells. After 6 h cocultivation, CAR‐T cells lysed more than 70% MM1.s cells (average 72.95%) and 8226 cells (average 76.65%) at a E:T ratio of 2:1, which was significantly higher than control T cells (average 18.85% in MM1.s and 15.52% in 8226 cells). For U266 cells, CD38‐CAR‐T cells killed about 30% (average 30.87%) tumor cells, which was only 8.87% in control T cells (Figure 3A).

For the determination of the antitumor effect at lower E:T ratios, T cells were activated by CD3/28 dynabeads before transduction. The activation markers were monitored. Data showed that T cells treated with CD3/28 dynabeads showed higher expression of CD69, CD25, and HLA‐DR (Figure 3C). CAR‐T cells generated from activated T cells were cocultured with MM cell lines. Surprisingly, even at E:T ratio of 1:100, CAR‐T cells showed a significant cytotoxicity against MM cells (average 23.31% in U266, 50.57% in MM1.s, and 56.77% in 8226) compared with control T cells with statistical significance (average 7.97% in U266, 11.74% in MM1.s, and 14.48% in 8226) (Figure 3B). While, CAR‐T cells generated from non‐activated T cells killed less MM1.s and 8226 cells at E:T ratio of 1:50 and 1:100 (average 39.28% in MM1.s and 45.77% in 8226 at E:T ratio of 1:50, average 33.64% in MM1.s and 39.48% in 8226 at E:T ratio of 1:100) (Figure 3D).

### 3.5 Cytokine release of CAR‐T cells

The release of inflammatory cytokines was detected using a CBA kit. After 24 h cocultivation of CAR‐T or control T cells and target MM cell lines, the cell supernatant was collected and incubated with specific capture beads and PE‐labeled antibodies. Then the concentrations of IL‐2, IL‐3, IL‐8, IL‐9, GM‐CSF, granzyme B, TNF‐α, and IFN‐γ were measured. Data showed that except the production of granzyme B by CAR‐T cells cocultured with U266 similar to control T cells, CD38‐CAR‐T cells stimulated by MM cell lines generated significantly increased level of IL‐2, IL‐3, IL‐8, IL‐9, GM‐CSF, granzyme B, IFN‐γ, and TNF‐α than control T cells (Figure 3F).

### 3.6 Effect of CD38‐CAR‐T cells on the treatment of MM in vivo

**FIGURE 4:**
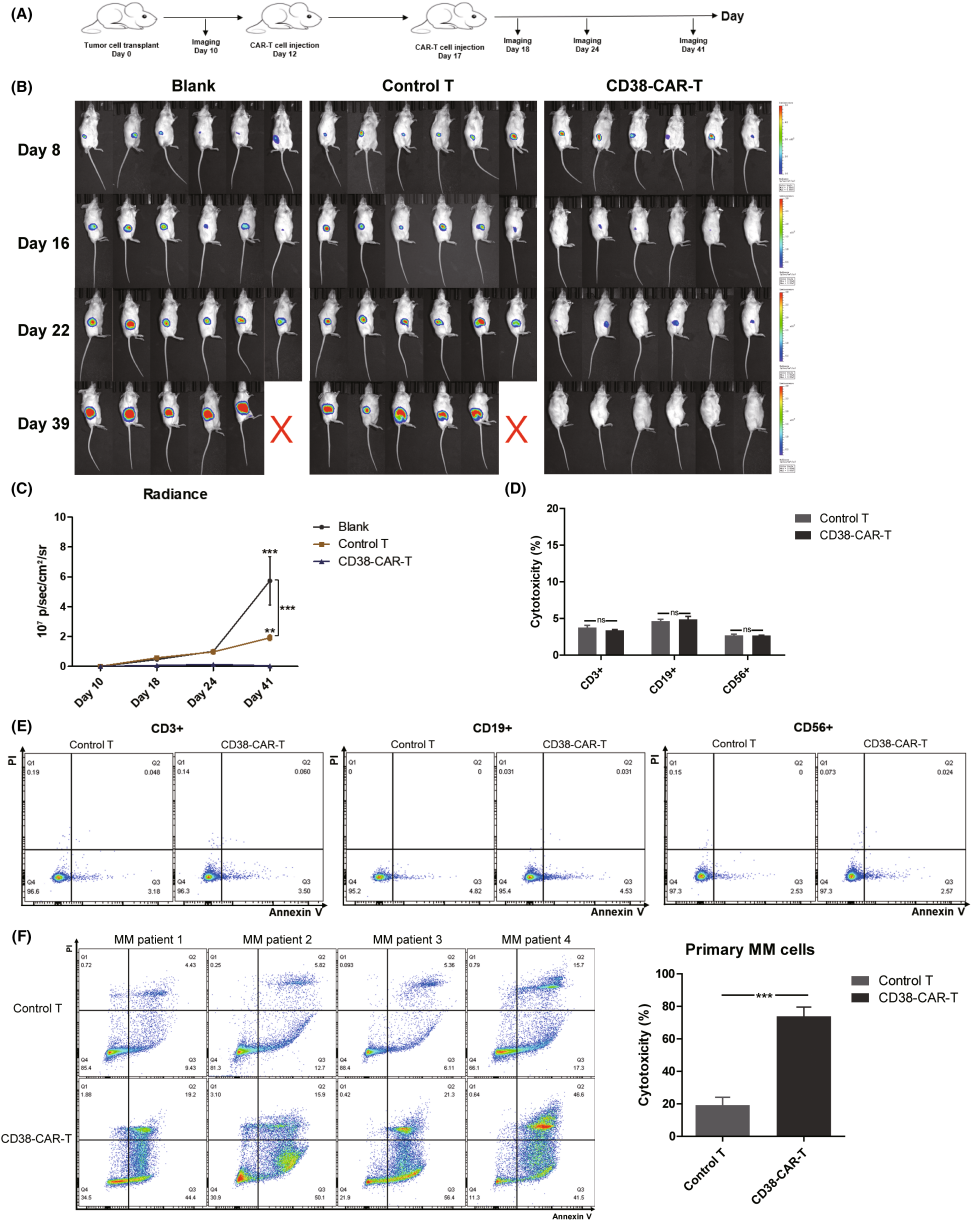
Effectiveness of CAR‐T cells on MM in vivo, PBMCs, and primary MM cells in vitro. (A) Overview of treatment of xenograft tumor model in vivo. (B, C) Tumor growth was monitored by IVIS living imaging. The mouse tumor burden was indicated by bioluminescence radiance. Mice treatment with CD38‐CAR‐T cells induced a significant antitumor effect compared to mice treated with control T cells or the blank group. While treatment of control T cells showed a certain therapeutic effect, tumor burden still progressed rapidly in the mice. (D, E) CD38‐CAR‐T cells showed limited cytotoxicity against CD3^+^, CD19^+^, and CD56^+^ subgroups of PBMCs. (F) CD38‐CAR‐T cells showed significant cytotoxicity against primary MM cells derived from patients. *p* < 0.05 was considered statistically significant. ns, not significant; *p < 0.05; **p < 0.01; ***p < 0.001.

In vitro studies showed that CAR‐T cells exhibited valid antitumor effect than control T cells. Hence, we further investigated the efficacy of CAR‐T cells against MM cells in subcutaneously tumor xenograft NSG mice models. Mice were treated with CAR‐T or control T cells. The tumor burden was monitored by luminescence intensity using the IVIS (Figure 4A).

Tumor growth showed aggressive progression of tumors in the untreated group. Compared to mice in the blank group, although treatment of control T cells showed a moderate therapeutic effect, the tumor progression was still rapid. While treatment with CAR‐T cells had a significant antitumor effect. Tumor burdens showed significant differences between CAR‐T‐cell treatment group and blank group or control T‐cell treatment group (Figure 4B,C).

### 3.7 Effect of CD38‐CAR‐T cells on autologous PBMCs and primary MM cells

Besides MM cells, CD38 also exists on the surface of normal hematopoietic and non‐hematopoietic tissues. This may result in tissue damage that is not intended for treatment. Then we investigated the effect of CAR‐T cells on autologous blood cells. CAR‐T or control T cells were co‐incubated with both autologous PBMCs for 24 h. Then T, B, and natural killer cells were labeled and detected by FACS. Results showed that CAR‐T cells showed limited cytotoxicity to T, B, and natural killer cells. (Figure 4D,E).

T cells and CD38^+^ primary tumor cells were isolated from the bone marrow of MM patients (Table 2). T cells were transduced and cocultured with primary MM cells at a E:T ratio of 1:10 for 24 h. CAR‐T cells showed an enhanced cytotoxicity against tumor cells (Figure 4F).

**TABLE 2:** Clinical data of MM patients.

No.	Sex	Age	Date of diagnosis	Therapeutic regimen	Biomarkers
Patient 4	F	56 years	2023.02.15	Bortezomib+ lenalidomide+ dexamethasone	CD38^+^ CD138^+^ BCMA^+^
Patient 1	F	71 years	2023.02.1	Bortezomib+ cyclophosphamide+ dexamethasone	CD38^+^ CD138^+^ BCMA^+^
Patient 3	M	61 years	2020.12.22	Bortezomib+ lenalidomide+ dexamethasone	CD38^+^ CD138^+^ BCMA^+^
Patient 2	F	57 years	2020.06.17	Bortezomib+ lenalidomide+ dexamethasone	CD38^+^ CD138^+^ BCMA^+^

## 4 DISCUSSION

CD38 molecule is highly expressed on MM cells, but is low expressed on normal tissues. MM progenitor cells were suggested to be enriched in CD38^+^ CD138^−^ subgroups.^18^ CD38^+^ MM cells in the minimal residual disease of bone marrow after autologous transplantation treatment may lead to reduced PFS in MM patients.^19^, ^20^ Hence, immunotherapy in MM targeting CD38 has drawn much attention. Daratumumab and isatuximab have shown efficacy in the treatment of MM. Daratumumab eliminates CD38^+^ MM cells by inducing Fc receptor‐mediated apoptosis, antibody‐dependent cellular cytotoxicity (ADCC) and complement‐dependent cytotoxicity (CDC).^28^ In addition, daratumumab can improve the antitumor immune response by eliminating CD38^+^ immunosuppressive cells.^29^ While isatuximab acts on tumor cells through lysosome‐dependent pro‐apoptotic activity and antibody‐dependent cellular phagocytosis (ADCP) besides ADCC and CDC.^28^ Isatuximab enhances the antitumor effect of T cells and NK cells by decreasing the number of regulatory T cells and production of IL‐10.^29^

In the past decade, a pioneering treatment, CAR‐T‐cell therapies have shown prominent antitumor effect in B‐cell malignancies.^21^, ^22^ Our previous study showed that CAR‐T cells targeting CD7 may be a promising strategy for T‐lymphoblastic leukemia/lymphoma.^23^ CAR‐T‐cell therapies in the administration of MM are in the early stage. BCMA is an ideal target due to its selective expression on mature B lymphocytes and plasma cells.^24^ However, MM cells often lost expression of BCMA upon recurrence of disease after first infusion of BCMA‐CAR‐T cells.^25^ CD138 is a specific biomarker for plasma cells. Reported data demonstrated the safety and modest therapeutic response of CD138‐CAR‐T cells.^25^, ^26^

In this study, second‐generation CD38‐CAR‐T cells were developed and effects of CAR‐T cells on MM cells were evaluated. Cocultured with MM cell lines, CD38‐CAR‐T cells showed increased expression of T‐cell activation markers in both CD4^+^ and CD8^+^ subgroups. Meanwhile, CD38^+^ MM cell lines significantly promoted the proliferation of CAR‐T cells compared with control T cells. However, the stimulation of CAR‐T cells and control T cells by MM cells seemed different. Cocultured with MM cell lines, CD8^+^ proportion of CAR‐T cells increased markedly. While in control T cells, CD4^+^ proportion is elevated. This may be due to the use of 4‐1BB as a costimulatory signal in the CAR construct. Studies have shown the principal role of 4‐1BB in the activation of CD8^+^ T cells rather than CD4^+^ T cells.^27^

Compared with control T cells, CAR‐T cells could eliminate MM cells more rapidly and efficiently. Notably, CD38‐CAR‐T cells killed about 40% U266 cells, which was consent with its low expression of CD38 (33.6% positive rate). With more than 90% positive rate of CD38 expression, over 85% MM1.s and 8226 cells were lysed by CAR‐T cells. These indicated that CAR‐T cells can recognize and eliminate tumor cells in an HLA nonindependent manner. Similar to the results in MM cell lines, CAR‐T cells also showed a significant cytotoxicity against CD38^+^ primary tumor cells isolated from MM patients. In vitro cytotoxicity of CAR‐T cells was verified by the results of in vivo mouse models. Tumor progressed rapidly in mice without CAR‐T‐cell treatment. However, CD38‐CAR‐T cell therapy significantly inhibited tumor growth.

Activated by target antigens, CAR‐T cells can release pro‐inflammatory cytokines and lysis tumor cells.^28^ In our study, CAR‐T cells produced higher level of cytokines such as IL‐2, IL‐3, IL‐8, IL‐9, GM‐CSF, granzyme B, IFN‐γ, and TNF‐α under the stimulation of MM cells. All these results indicated the robust anti‐tumor activity of CD38‐CAR‐T cells against MM cells, even though CD38 was not expressed on transduced CAR‐T cells.

Except plasma cells, CD38 was also expressed at lower levels on a fraction of normal cells and tissues.^14^ This may arouse concern about organic damage risk of CD38‐targeting immunotherapy. According to data of the Human Protein Profile Database (https://www.proteinatlas. org/), CD38 was only found strongly positive expression in prostate, lymph node, and tonsil. This is consistent with our IHC results. We further assessed the effect of CAR‐T cells on autologous PBMCs. CAR‐T cells showed no obvious cytotoxicity against T, B, and natural killer cells. Studies have also indicated the safety of immunotherapy targeting CD38 molecules. Drent et al. demonstrated that proliferation of blood progenitor cells was not inhibited by CD38‐CAR‐T cells.^14^ An et al. reported a slight cytotoxicity of CD38‐CAR‐T cells against CD38^+^ blood cells.^29^ Daratumumab treatment also showed little or no toxicity for prolonged administration periods.^8^ However, cytotoxicity of CAR‐T cells against CD38^+^ cells in non‐hematopoietic tissues could not be evaluated. Optimization of the tumor cell affinity of CAR‐T cells may improve the specificity for CD38 recognizing and reduce side effects.

Studies of CAR‐T cells targeting CD38 have demonstrated their anti‐tumor efficacy. The effective clearance of tumor cells by CD38‐CAR‐T cells requires a longer time or higher E:T ratios.^18^, ^19^, ^20^ In our study, CAR‐T cells generated from CD3/28 dynabeads activated T cells showed a significant cytotoxicity activity on MM1.s and RPMI8226 cells in a short period of 24 h and low E:T ratios.

In this study, a CD38‐CAR‐T cell was constructed and exhibited robust cytolysis against MM cell lines. We first demonstrated the dynamic changes of activation markers and more cytokines generated by CAR‐T cells with the stimulation of MM cells. It may provide a promising and effective solution for the treatment of MM and even CD38^+^ malignancies. Further clinical trials are required to evaluate its efficacy and safety.

## AUTHOR CONTRIBUTIONS

**Hongwen Li:** Project administration (lead); writing – original draft (lead). **Jing Li:** Project administration (supporting). **Jiazhuo Wu:** Project administration (supporting). **Zhuangzhuang Shi:** Project administration (supporting). **Yuyang Gao:** Project administration (supporting). **Wenting Song:** Writing – review and editing (equal). **Jiwei Li:** Writing – review and editing (equal). **Zhaoming Li:** Writing – review and editing (equal). **Mingzhi Zhang:** Conceptualization (lead); supervision (equal); writing – review and editing (equal).

## FUNDING INFORMATION

This work was supported by the National Natural Science Foundation of China (81970184; 82170183; U1904139; 82070209).

## CONFLICT OF INTEREST STATEMENT

The authors declare no conflicts of interest.

## Supporting information


Supplementary Figure 1

## Data Availability

Data sharing is not applicable to this article as no new data were created or analyzed in this study.

## References

[1] Maude SL , Laetsch TW , Buechner J , et al. Tisagenlecleucel in children and young adults with B‐cell lymphoblastic leukemia. N Engl J Med. 2018;378:439‐448. doi:10.1056/NEJMoa1709866 29385370 PMC5996391

[2] Schuster SJ , Svoboda J , Chong EA , et al. Chimeric antigen receptor T cells in refractory B‐cell lymphomas. N Engl J Med. 2017;377:2545‐2554. doi:10.1056/NEJMoa1708566 29226764 PMC5788566

[3] Raje N , Berdeja J , Lin Y , et al. Anti‐BCMA CAR T‐cell therapy bb2121 in relapsed or refractory multiple myeloma. N Engl J Med. 2019;380:1726‐1737. doi:10.1056/NEJMoa1817226 31042825 PMC8202968

[4] Zhao WH , Liu J , Wang BY , et al. A phase 1, open‐label study of LCAR‐B38M, a chimeric antigen receptor T cell therapy directed against B cell maturation antigen, in patients with relapsed or refractory multiple myeloma. J Hematol Oncol. 2018;11:141. doi:10.1186/s13045-018-0681-6 30572922 PMC6302465

[5] Zhang WY , Wang Y , Guo YL , et al. Treatment of CD20‐directed chimeric antigen receptor‐modified T cells in patients with relapsed or refractory B‐cell non‐Hodgkin lymphoma: an early phase IIa trial report. Signal Transduct Target Ther. 2016;1:16002. doi:10.1038/sigtrans.2016.2 29263894 PMC5661644

[6] Tan Y , Cai H , Li C , et al. A novel full‐human CD22‐CAR T cell therapy with potent activity against CD22(low) B‐ALL. Blood Cancer J. 2021;11:71. doi:10.1038/s41408-021-00465-9 33839735 PMC8036232

[7] Turtle CJ , Hay KA , Hanafi LA , et al. Durable molecular remissions in chronic lymphocytic leukemia treated with CD19‐specific chimeric antigen receptor‐modified T cells after failure of ibrutinib. J Clin Oncol. 2017;35:3010‐3020. doi:10.1200/JCO.2017.72.8519 28715249 PMC5590803

[8] Malavasi F , Deaglio S , Funaro A , et al. Evolution and function of the ADP ribosyl cyclase/CD38 gene family in physiology and pathology. Physiol Rev. 2008;88:841‐886. doi:10.1152/physrev.00035.2007 18626062

[9] Lokhorst HM , Plesner T , Laubach JP , et al. Targeting CD38 with daratumumab monotherapy in multiple myeloma. N Engl J Med. 2015;373:1207‐1219. doi:10.1056/NEJMoa1506348 26308596

[10] Mikhael J , Richter J , Vij R , et al. A dose‐finding phase 2 study of single agent isatuximab (anti‐CD38 mAb) in relapsed/refractory multiple myeloma. Leukemia. 2020;34:3298‐3309. doi:10.1038/s41375-020-0857-2 32409691 PMC7685976

[11] Mikkilineni L , Kochenderfer JN . Chimeric antigen receptor T‐cell therapies for multiple myeloma. Blood. 2017;130:2594‐2602. doi:10.1182/blood-2017-06-793869 28928126 PMC5731088

[12] Gagelmann N , Riecken K , Wolschke C , et al. Development of CAR‐T cell therapies for multiple myeloma. Leukemia. 2020;34:2317‐2332. doi:10.1038/s41375-020-0930-x 32572190

[13] Danylesko I , Beider K , Shimoni A , Nagler A . Novel strategies for immunotherapy in multiple myeloma: previous experience and future directions. Clin Dev Immunol. 2012;2012:753407. doi:10.1155/2012/753407 22649466 PMC3357929

[14] Drent E , Groen RW , Noort WA , et al. Pre‐clinical evaluation of CD38 chimeric antigen receptor engineered T cells for the treatment of multiple myeloma. Haematologica. 2016;101:616‐625. doi:10.3324/haematol.2015.137620 26858358 PMC5004365

[15] Partida‐Sanchez S , Cockayne DA , Monard S , et al. Cyclic ADP‐ribose production by CD38 regulates intracellular calcium release, extracellular calcium influx and chemotaxis in neutrophils and is required for bacterial clearance in vivo. Nat Med. 2001;7:1209‐1216. doi:10.1038/nm1101-1209 11689885

[16] Partida‐Sanchez S , Goodrich S , Kusser K , Oppenheimer N , Randall TD , Lund FE . Regulation of dendritic cell trafficking by the ADP‐ribosyl cyclase CD38: impact on the development of humoral immunity. Immunity. 2004;20:279‐291. doi:10.1016/s1074-7613(04)00048-2 15030772

[17] Reddy M , Eirikis E , Davis C , Davis HM , Prabhakar U . Comparative analysis of lymphocyte activation marker expression and cytokine secretion profile in stimulated human peripheral blood mononuclear cell cultures: an in vitro model to monitor cellular immune function. J Immunol Methods. 2004;293:127‐142. doi:10.1016/j.jim.2004.07.006 15541283

[18] Hosen N , Matsuoka Y , Kishida S , et al. CD138‐negative clonogenic cells are plasma cells but not B cells in some multiple myeloma patients. Leukemia. 2012;26:2135‐2141. doi:10.1038/leu.2012.80 22430638

[19] Liu H , Yuan C , Heinerich J , et al. Flow cytometric minimal residual disease monitoring in patients with multiple myeloma undergoing autologous stem cell transplantation: a retrospective study. Leuk Lymphoma. 2008;49:306‐314. doi:10.1080/10428190701813018 18231918

[20] Atanackovic D , Steinbach M , Radhakrishnan SV , Luetkens T . Immunotherapies targeting CD38 in multiple myeloma. Onco Targets Ther. 2016;5:e1217374. doi:10.1080/2162402X.2016.1217374 PMC5139636 27999737

[21] Ormhoj M , Bedoya F , Frigault MJ , Maus MV . CARs in the lead against multiple myeloma. Curr Hematol Malig Rep. 2017;12:119‐125. doi:10.1007/s11899-017-0373-2 28233151 PMC5410390

[22] Kohl U , Arsenieva S , Holzinger A , Abken H . CAR T cells in trials: recent achievements and challenges that remain in the production of modified T cells for clinical applications. Hum Gene Ther. 2018;29:559‐568. doi:10.1089/hum.2017.254 29620951

[23] Zhang M , Chen D , Fu X , et al. Autologous Nanobody‐derived fratricide‐resistant CD7‐CAR T‐cell therapy for patients with relapsed and refractory T‐cell acute lymphoblastic leukemia/lymphoma. Clin Cancer Res. 2022;28:2830‐2843. doi:10.1158/1078-0432.CCR-21-4097 35435984

[24] Carpenter RO , Evbuomwan MO , Pittaluga S , et al. B‐cell maturation antigen is a promising target for adoptive T‐cell therapy of multiple myeloma. Clin Cancer Res. 2013;19:2048‐2060. doi:10.1158/1078-0432.CCR-12-2422 23344265 PMC3630268

[25] Teoh PJ , Chng WJ . CAR T‐cell therapy in multiple myeloma: more room for improvement. Blood Cancer J. 2021;11:84. doi:10.1038/s41408-021-00469-5 33927192 PMC8085238

[26] Kegyes D , Constantinescu C , Vrancken L , et al. Patient selection for CAR T or BiTE therapy in multiple myeloma: which treatment for each patient? J Hematol Oncol. 2022;15:78. doi:10.1186/s13045-022-01296-2 35672793 PMC9171942

[27] Etxeberria I , Glez‐Vaz J , Teijeira A , Melero I . New emerging targets in cancer immunotherapy: CD137/4‐1BB costimulatory axis. ESMO Open. 2020;4:e000733. doi:10.1136/esmoopen-2020-000733 32611557 PMC7333812

[28] Bonifant CL , Jackson HJ , Brentjens RJ , Curran KJ . Toxicity and management in CAR T‐cell therapy. Mol Ther Oncolytics. 2016;3:16011. doi:10.1038/mto.2016.11 27626062 PMC5008265

[29] An N , Hou YN , Zhang QX , et al. Anti‐multiple myeloma activity of nanobody‐based anti‐CD38 chimeric antigen receptor T cells. Mol Pharm. 2018;15:4577‐4588. doi:10.1021/acs.molpharmaceut.8b00584 30185037

