# Supplementary figures and images for: A second‐generation CD38‐CAR‐T cell for the treatment of multiple myeloma
Source: Cancer Med. 2023 Apr 11;12(9):10804–15. doi: 10.1002/cam4.5818 (PMC10225187; doi:10.1002/cam4.5818)

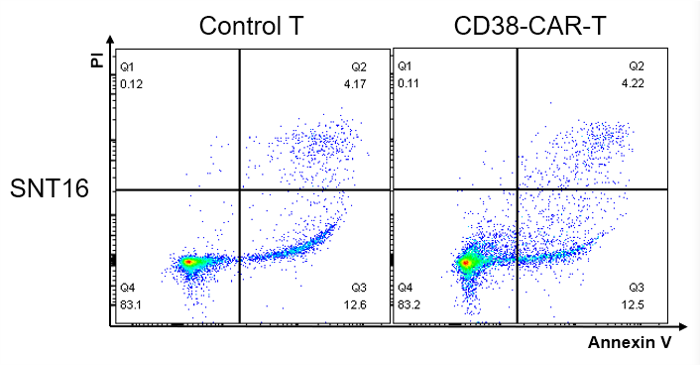

Supplement: Supplementary file 1 — Supplementary Figure 1 [file CAM4-12-10804-s001.tif]
